# Case Report: Early diagnosis and bevacizumab-based chemotherapy for primary pericardial mesothelioma: a case with occupational asbestos exposure history

**DOI:** 10.3389/fcvm.2023.1257373

**Published:** 2023-11-20

**Authors:** Daniel Wang, Yung Hsuan Wang, Sung Chao Chu

**Affiliations:** ^1^ The School of Medicine, Tzu Chi University, Hualien, Taiwan; ^2^ Faculty of Medicine, Umea University, Umea, Sweden; ^3^ Division of Chest Medicine, Hualien Tzu Chi Hospital, Buddhist Tzu Chi Medical Foundation, Hualien, Taiwan; ^4^ Department of Hematology and Oncology, Hualien Tzu Chi Hospital, Buddhist Tzu Chi Medical Foundation, Hualien, Taiwan

**Keywords:** primary pericardial mesothelioma, asbestos, occupational exposure, bevacizumab, chemotherapy

## Abstract

**Background:**

Primary pericardial mesothelioma (PPM) is an exceedingly rare malignant cancer and has a poor prognosis, which has been partly attributed to its frequently delayed diagnosis due to its nonspecific syndromes, its similar presentation to benign pericardial diseases, and its non-definitive etiology. In many PPM cases, the time from presentation to definite diagnosis may last for several months or even over one year. Unlike pleural mesothelioma, the relationship between PPM and asbestos exposure remains unsettled. To date, there is no consensus on the treatment of PPM.

**Case report:**

The patient is a 57-year-old male who had nonspecific syndromes and inconclusive image findings. The occupational long-term asbestos exposure history of this patient raised our concerns regarding potential malignancy when confronted with unexplained pericardial effusion accompanied by cardiac tamponade. The heightened suspicion prompted us to perform pericardiocentesis and biopsy on the third day after admission to our department. An early diagnosis of PPM was established by the pathological and immunohistochemical evaluation of the biopsy specimen two weeks after admission. Positron emission tomography-computed tomography revealed that the lesion was localized at the anterior part of the mediastinum without distant metastasis. This patient refused to receive cardiac surgery. He subsequently underwent six cycles of chemotherapy (cisplatin plus pemetrexed) in combination with bevacizumab (a humanized anti-VEGF antibody) as the first-line treatment, resulting in complete relief of symptoms and satisfactory outcomes with no complications. Four months after the first course, the patient initiated a second course of chemotherapy with a similar regimen, but he opted to discontinue the medical treatment after the initiation of the second course. The patient was transferred to the hospice care unit and unfortunately expired one year after the initial presentation.

**Conclusion:**

We present a case of an early multidisciplinary clinical approach to diagnose and manage PPM with consideration of occupational asbestos exposure history and clinical symptoms. Bevacizumab-based chemotherapy remains an option for the treatment of PPM.

## Introduction

Primary pericardial mesothelioma (PPM) is an exceedingly rare malignant cancer accounting for only 0.7% of all malignant mesotheliomas, with an annual standardized incidence rate of approximately 0.36 per 10 million person-years (1–5). PPM has a poor prognosis with a median survival of less than six months (1, 4–6), which has been partly attributed to its frequently delayed diagnosis (6–8). In many PPM cases, the time from presentation to definite diagnosis may last for several months (4–7) or even over one year (6, 9–14). This slow recognition is likely due to its nonspecific syndromes, its similar presentation to benign pericardial diseases, and its non-definitive etiology (1, 6–8, 10–15). It has been suggested that early detection of this disease is the only hope for survival (2). Unlike pleural mesothelioma, the relationship between PPM and asbestos exposure remains unsettled; some investigators reported an association with asbestos exposure (3, 4, 16), while others reported no or weak correlation (6–10, 12–15, 17–19). So far, there is no consensus on the treatment of PPM, although the survival benefit of chemotherapy has been shown to be superior to that of surgery (6). In almost all reported cases with chemotherapy, a doublet regimen, cisplatin plus pemetrexed, was used as the first-line treatment (6, 7, 9–11, 17, 18, 20, 21). Here we present a case of early diagnosis of PPM promoted by the indication of the patient's occupational asbestos exposure history and clinical presentations. This patient was subsequently treated with bevacizumab (a humanized anti-VEGF antibody) (2) combined with first-line chemotherapy.

### Case presentation

A 57-year-old male patient who presented with left-sided chest tightness, frequent dry cough, progressive orthopnea, and a weight loss of 5 kg over a 4-month period was admitted to our department on 18th July 2022 (Day 0). The patient complained that he began to feel chest tightness and dry coughing at night about a month. He had an unremarkable medical history, was an active smoker consuming 1 pack of cigarettes every 3 days and had a 20-year history of occupational asbestos exposure from his work as an interior designer. He frequently came into contact with sound and heat insulation material containing high levels of asbestos and did not consistently use a face mask.

**Figure 1:**
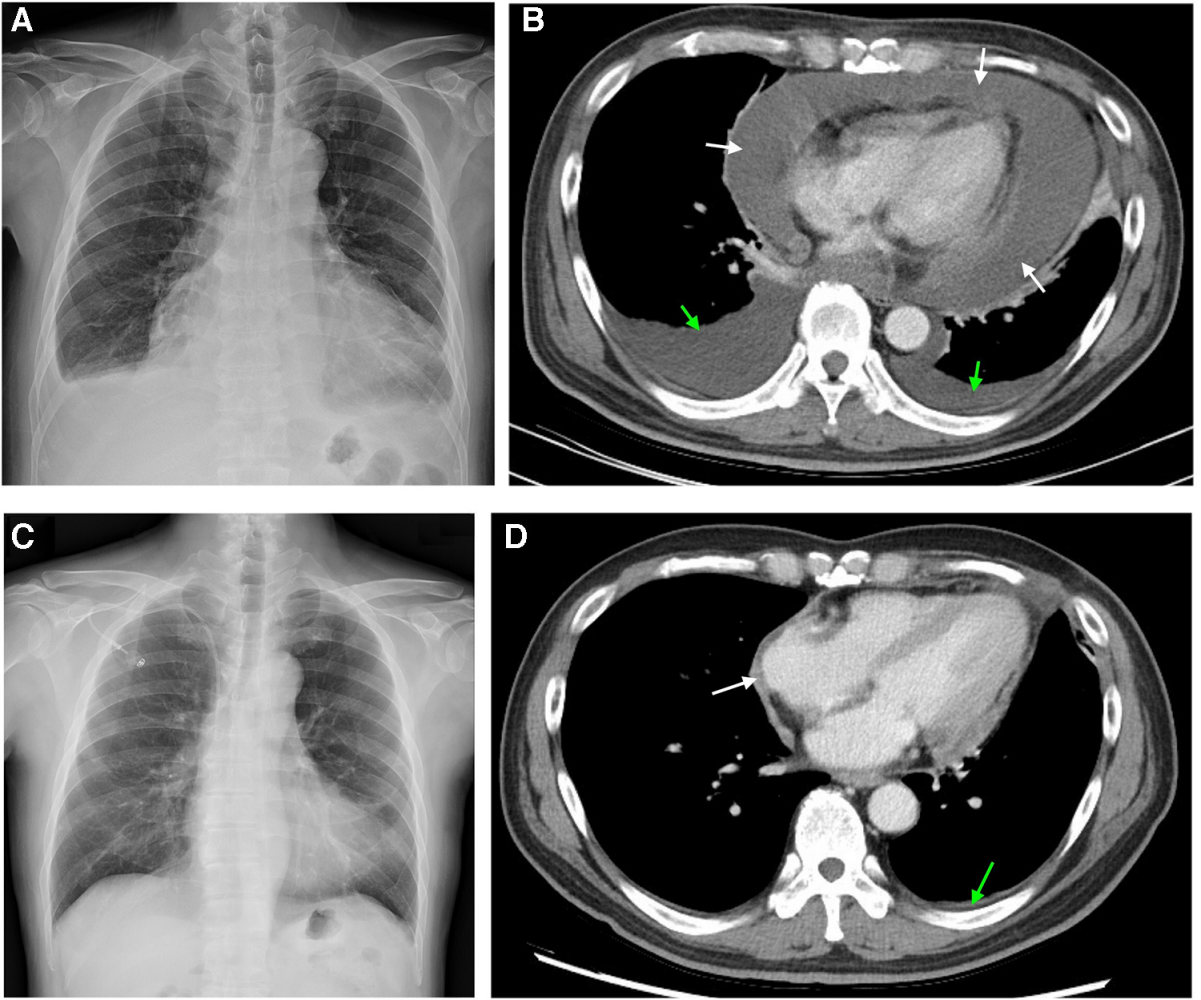
Chest image findings before and after 6 cycles of chemotherapy. (**A**) x-ray image shows cardiomegaly with pericardial effusion and blunted left costophrenic angle. (**B**) Computed tomography image shows pericardial effusion with noticeable pericardial thickening and bilateral pleural effusion. (**C**) x-ray image showed normal heart size without pleural effusion. (**D**) Computed tomography image showed subsiding pericardial effusion and lessened pericardial thickening. White and green arrows indicate the areas of pericardial and pleural effusion, respectively.

The patient's body weight and length were 73.4 kg and 173 cm, respectively, and his blood pressure, heart rate, and respiratory rate were 102/67 mmHg, 81 beats/minute, and 24 breaths/minute, respectively. The physical examination was notable for bilateral pitting edema of the lower legs. Muffled and irregular heart sounds were detected with jugular vein elevation. Electrocardiography showed sinus tachycardia without abnormal T waves or ST segment changes. The initial laboratory analysis revealed that the white blood cell count was 16,390 cells/mm^3^, the BUN/creatinine ratio was 31/1.16, the high sensitivity troponin I level (hs-troponin I) was 4.3 pg/ml, NT-proBNP was 418.0 pg/ml, and carcinoembryonic antigen (CEA) was 1.9 ng/ml. Chest x-ray showed cardiomegaly and pericardial effusion (Figure 1A). Computed tomography (CT) scans revealed massive pericardial effusion with thickened pericardium and bilateral pleural effusion (Figure 1B).

**Figure 2:**
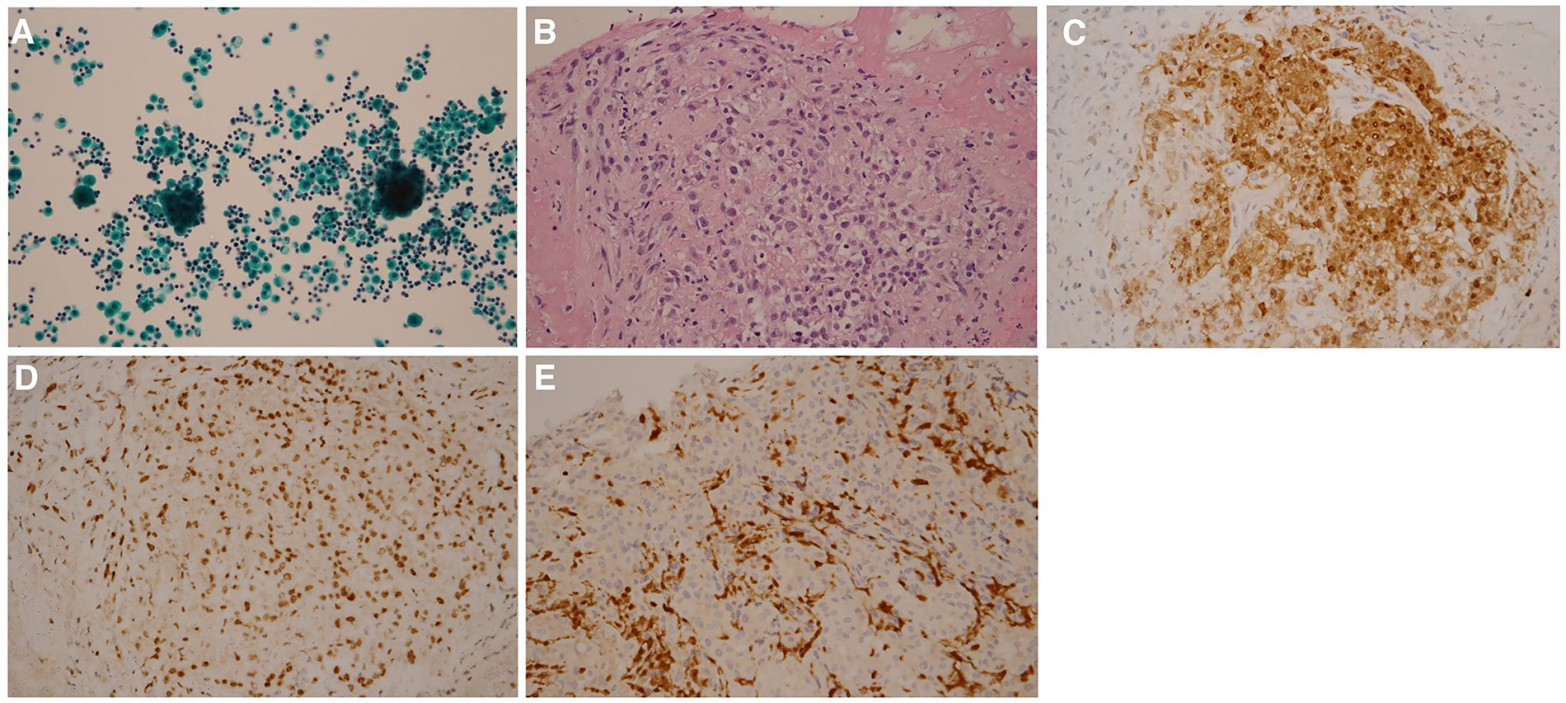
Pathology findings. Cytopathology analysis of pericardial effusion shows clustered mesothelial cells (**A**) H&E stain analysis of pericardial biopsy shows atypical mesothelioma cells (**B**) Immunohistological analysis of pericardial biopsy shows that tumor cells exhibited positive staining of cytokeratin 5/6 (**C**), retained expression of BRCA1-associated protein 1 (**D**), and loss of expression of methylthioadenosine phosphorylase (**E**) Magnifications: 10x in panel (**A**), 40x in panel (**B**), and 20x in panels (**C**–**E**).

**Figure 3:**
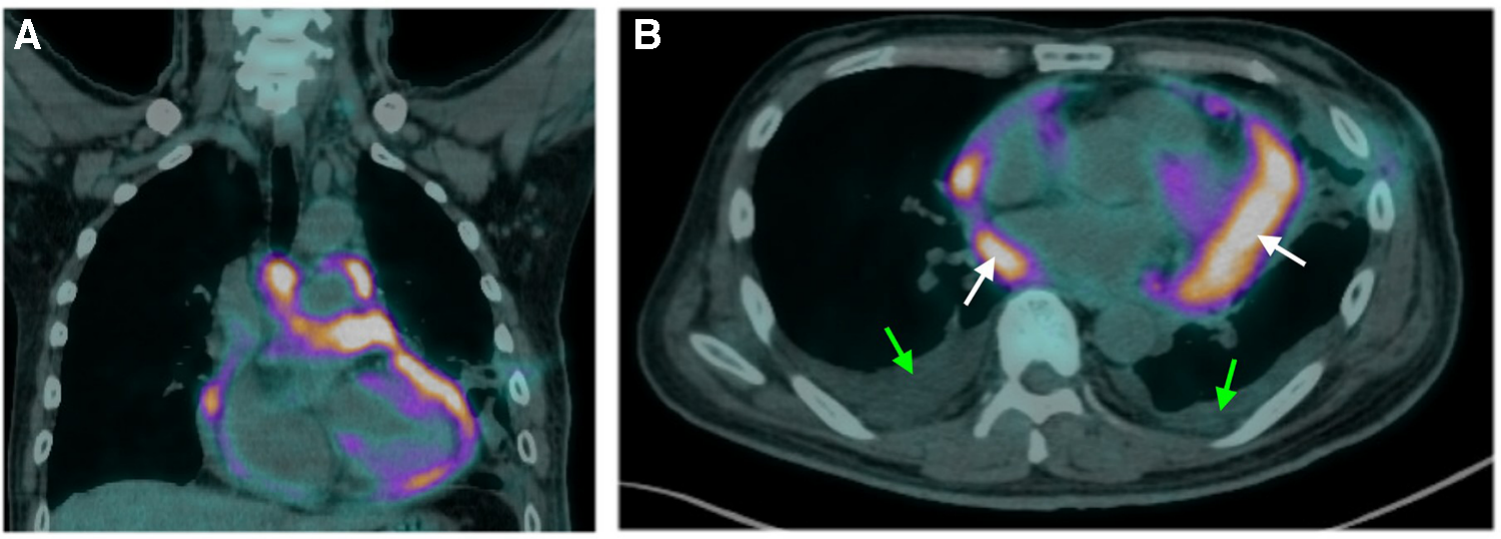
Findings of positron emission tomography-computed tomography. Sagittal (**A**) and axial (**B**) images illustrate diffuse, irregular nodular uptake of fluorodeoxyglucose in the anterior mediastinum with no metastasis. White and green arrows indicate the areas of pericardial and pleural effusion, respectively.

Pericardiocentesis was performed on Day 1 to relieve the symptoms of the unexplained pericardial effusion, and the drainage of pericardiocentesis came out with a purulent and bloody effusion. In light of his history of occupational long-term asbestos exposure and clinical data, a thoracoscopic pericardial biopsy was conducted on Day 3. Subsequent cytopathology analysis of pericardial effusion showed clustered mesothelial cells (Figure 2A). Pathological evaluation of the biopsy specimen revealed the presence of several abnormalities, including an overgrowth of mesothelial cells, changes in the nuclei of these cells, increased collagen in the connective tissue, and the accumulation of fibrinoid exudates (Figure 2B). Immunohistochemistry shows positive mesothelial 183 markers (cytokeratin 5/6 and calretinin) and negative pulmonary 184 epithelial markers (thyroid transcription factor-1 and 185 carcinoembryonic antigen). The tumor cells also exhibited a loss of 186 expression of methylthioadenosine phosphorylase and retained 187 expression of BRCA1-associated protein 1 (Figure 2C–2E). Positron emission tomography-computed tomography (PET-CT) was then performed to detect potential metastasis on Day 10. The result showed 18F-fluorodeoxyglucose (FDG) uptake in the anterior part of the mediastinum without distant metastasis (Figure 3). With these pathological findings, the diagnosis of PPM was made on Day 11.

**Figure 4:**
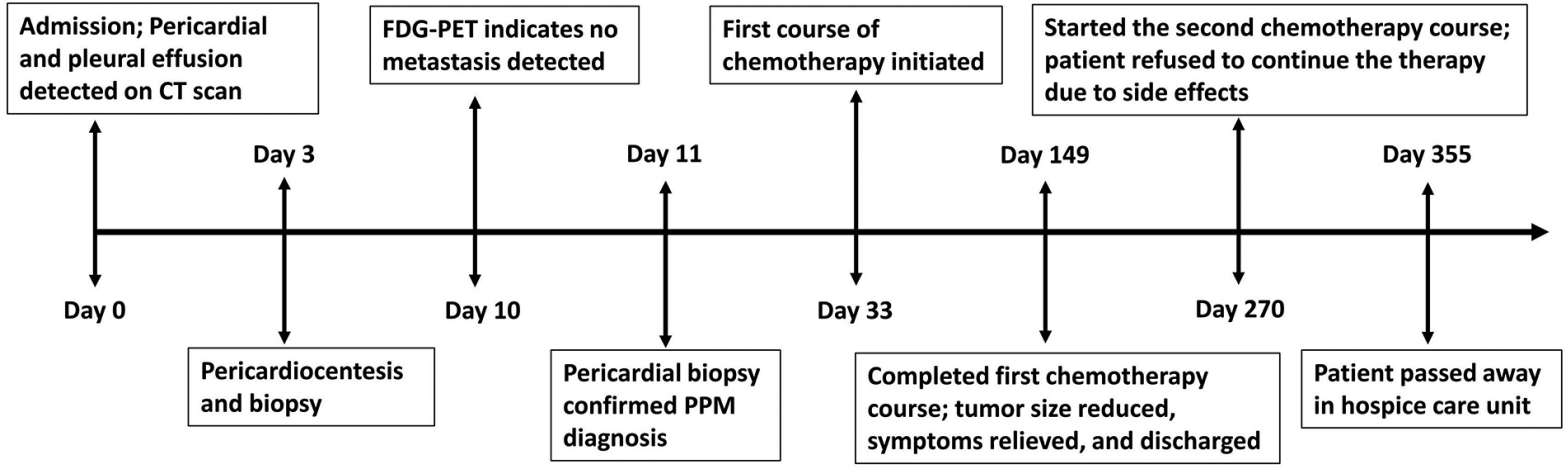
Timeline summarizing the major events during the episode of care for the patient. CT, computed tomography; FDG, 18F-fluorodeoxyglucose; PPM, primary pericardial mesothelioma.

After the diagnosis, our patient was recommended to receive a combined cardiac tumor debulking surgery and palliative chemotherapy, but he opted for palliative chemotherapy only, which started on Day 33. For cycles 1–6, the patient was administered Avastin (bevacizumab) at a dose of 5 mg/kg, Cisplatin at a dose of 50–60 mg/m^2^, and Alimta (Pemetrexed) at a dose of 400–500 mg/m^2^. The patient tolerated the first cycle well and experienced mild appetite loss and nausea. The patient received acupuncture as an adjuvant therapy aiming to minimize the treatment-related symptoms. There were no other treatment-related side effects reported. A follow-up chest x-ray and CT scan immediately after the 6 cycles of chemotherapy on Day 149 revealed normal heart size, no pleural or pericardial effusion (Figure 1C), and lessened pericardial thickening (Figure 1D). The follow-up laboratory analysis revealed that the white blood cell count was 5.89 cells/mm^3^, the BUN/creatinine ratio was 15/1.03, the hs-troponin I was 5.8 ng/ml, NT-proBNP was 871 pg/ml, and CEA was 1.6 ng/ml. Subsequently, the patient was discharged with minimal side effects, such as mild body weight loss, appetite loss, and fatigue. On Day 270, the patient initiated a second course of chemotherapy due to an escalation in episodes of arrhythmia and mild dyspnea. However, the patient opted to discontinue the medical treatment after the initiation of the second course due to side effects. We did fully discuss with the patient and family members regarding the treatment. The patient also received mental consultation from experts. The patient nevertheless refused to receive further treatment. Subsequently, the patient was transferred to the hospice care unit and unfortunately expired on Day 355. The timeline of the major events during the episode of care for this patient is summarized in Figure 4.

## Discussion

This report describes a patient with PPM who had an occupational long-term asbestos exposure history and who received chemotherapy only after diagnosis resulting in satisfactory outcomes. In this case, early diagnosis of PPM and bevacizumab-based chemotherapy are two main points that deserve to be highlighted.

Our patients underwent a total of 6 cycles of chemotherapy in the first course with a combination of bevacizumab as the first-line treatment, resulting in complete relief of symptoms and satisfactory outcomes. This choice was made based on the patient's preference for palliative chemotherapy over surgical intervention following a full discussion of treatment plans. Due to its rarity, there is no consensus on the treatment of PPM (1, 2, 5, 6). Currently, treatment options for PPM are adapted from the more often studied diffuse pleural mesotheliomas (1, 2, 18); surgery is the most widely-used approach, followed by chemotherapy (5, 6, 18). However, in a review of 103 published PPM cases, it was found that chemotherapy, but not surgery, provided a statistically significant survival benefit. Of note, a doublet regimen, cisplatin plus pemetrexed, was used as the first-line treatment in almost all reported cases with chemotherapy (6, 7, 9–11, 17, 18, 20, 21). In only one reported case (14), a combination of bevacizumab, cisplatin, and pemetrexed was used as the first-line treatment for PPM, but unfortunately, the tumor remained stable after eight cycles of chemotherapy. Bevacizumab is a humanized anti-VEGF antibody that inhibits angiogenesis (2). In our case, the patient well tolerated the first course of chemotherapy in combination with bevacizumab with a satisfactory outcome. Unfortunately, the patient opted to discontinue the medical treatment after the initiation of the second course and subsequently expired. The use of bevacizumab has been shown to be promising in the treatment of malignant pleural mesothelioma (22). The addition of bevacizumab to standard-of-care chemotherapy has provided a novel therapeutic option in a range of advanced cancers (23). Several randomized controlled trials have been conducted to investigate its efficacy in different types of cancers, including colorectal cancer, lung cancer, breast cancer, renal cell carcinoma, cervical cancer, glioblastoma, and ovarian cancer (23). In light of this fact, we still suggest that bevacizumab may also be considered as an option for the treatment of PPM.

The diagnosis of PPM remains challenging, which leads to the situation that the diagnosis is usually made after surgery or at autopsy (2, 10, 15, 18, 21). The delayed diagnosis of PPM may be due to its nonspecific syndromes, its similar presentation to benign pericardial diseases, and its non-definitive etiology (1, 6–8, 10–15). Our patient also initially had nonspecific symptoms and inconclusive image findings. However, the occupational long-term asbestos exposure history of this patient raised our concerns regarding potential malignancy when confronted with unexplained pericardial effusion accompanied by cardiac tamponade. As a result, from the time of admission, it only took 11 days for us to establish a definitive diagnosis. The etiology of PPM is unclear. The relationship between PPM and asbestos exposure remains controversial (3, 4, 6–10, 12–19). Our finding regarding the early detection of PPM supports the notion that asbestos exposure plays a role in the pathogenesis of PPM. Although the mechanisms underlying this pathogenesis remain unclear, it has been proposed (24) that, after asbestos fibers are inhaled deeply into the lung and penetrate the pleural space, the interaction of asbestos fibers with mesothelial cells and inflammatory cells is thought to initiate prolonged cycles of tissue damage, repair, and local inflammation, which finally lead to carcinogenesis of malignant mesothelioma.

In conclusion, we present a case of an early multidisciplinary clinical approach to diagnose and manage PPM with consideration of occupational asbestos exposure history and clinical symptoms. Although the patient expired after the premature discontinuation of the second course of chemotherapy based on the patient's own decision, bevacizumab-based chemotherapy remains an option for the treatment of PPM.

## Data Availability

The original contributions presented in the study are included in the article/Supplementary Material, further inquiries can be directed to the corresponding author.
